# Online Natural Myocontrol of Combined Hand and Wrist Actions Using Tactile Myography and the Biomechanics of Grasping

**DOI:** 10.3389/fnbot.2020.00011

**Published:** 2020-02-27

**Authors:** Mathilde Connan, Risto Kõiva, Claudio Castellini

**Affiliations:** ^1^German Aerospace Center (DLR), Institute of Robotics and Mechatronics, Wessling, Germany; ^2^Research Institute Cognitive Interaction Technology, Bielefeld University, Bielefeld, Germany

**Keywords:** myocontrol, tactile myography, prosthetics, combined actions, grip strength, high-density force myography (HD-FMG), biomechanics of grasping

## Abstract

**Objective:** Despite numerous recent advances in the field of rehabilitation robotics, simultaneous, and proportional control of hand and/or wrist prostheses is still unsolved. In this work we concentrate on myocontrol of combined actions, for instance power grasping while rotating the wrist, by only using training data gathered from single actions. This is highly desirable since gathering data for all possible combined actions would be unfeasibly long and demanding for the amputee.

**Approach:** We first investigated physiologically feasible limits for muscle activation during combined actions. Using these limits we involved 12 intact participants and one amputee in a Target Achievement Control test, showing that tactile myography, i.e., high-density force myography, solves the problem of combined actions to a remarkable extent using simple linear regression. Since real-time usage of many sensors can be computationally demanding, we compare this approach with another one using a reduced feature set. These reduced features are obtained using a fast, spatial first-order approximation of the sensor values.

**Main results:** By using the training data of single actions only, i.e., power grasp or wrist movements, subjects achieved an average success rate of 70.0% in the target achievement test using ridge regression. When combining wrist actions, e.g., pronating and flexing the wrist simultaneously, similar results were obtained with an average of 68.1%. If a power grasp is added to the pool of actions, combined actions are much more difficult to achieve (36.1%).

**Significance:** To the best of our knowledge, for the first time, the effectiveness of tactile myography on single and combined actions is evaluated in a target achievement test. The present study includes 3 DoFs control instead of the two generally used in the literature. Additionally, we define a set of physiologically plausible muscle activation limits valid for most experiments of this kind.

## 1. Introduction

The umbrella term *myocontrol* denotes, in contemporary assistive robotics, the control of a mechatronic device exerted by a human subject using coordinated muscle contractions. Informally, it is about *reliably* turning patterns of biological signals into “actions,” usually to be executed by a prosthetic device, such as e.g., a “power grasp” or the “flexion of the wrist.” In this framework, *reliably* means that the prosthesis should be able to execute what the amputee desires, exactly when he/she desires it, and in a transparent (natural) way. To this aim, the keywords *natural control* (Castellini et al., 2014; Ortiz-Catalan and Branemark, 2014) and *simultaneous and proportional control* (Jiang et al., 2009; Muceli et al., 2014) have appeared in literature, denoting continuous, real-time, and graded control over many degrees of freedom (DoF) of the prosthesis—potentially, all of them. This idea is particularly well illustrated in Fougner et al. (2012) (consider **Figure 2** in the paper), which dates back to 2012.

There are multiple reasons why decades of academic research have yet hardly turned into commercial solutions (two remarkable exceptions are the *Complete Control* system by CoApt Engineering^1^ and *Myo Plus* by Ottobock^2^), the most prominent among which is, probably, the lack of reliability of said form of control. At the time of writing this article, seven years have gone by since the appearance of Jiang et al. (2012), a paper in which the community of myocontrol was incited, among other things, to find novel sensing techniques for intent detection. The traditional biosignals used in myocontrol, surface electromyography (sEMG), are deemed to be insufficient, and the research community is underway of finding alternatives. At present, no widely accepted and exhaustively tested alternative exists. We argue that a prominent option could likely be *tactile/force myography*. Since Craelius et al.'s experiments in the early 2000s (Curcie et al., 2001; Craelius, 2002) it has been clear that each pattern of muscle activation corresponding to a desired action also corresponds to a specific, repeatable pattern of external forearm pressure produced by the volumetric variation of the underlying muscles. Such a deformation could be detected by force/pressure sensors and associated to the action, thereby used as an alternative or parallel technique to surface electromyography. Examples of comparisons and mixtures of the two techniques can already be found in literature (Fang et al., 2015; Cho et al., 2016; Connan et al., 2016; Castellini et al., 2018). This approach, involving several independent force sensors has been called, among other ways, *force myography* or FMG.

In this work, we try to advance the state of the art in the usage of a closely related technique for myocontrol, namely *tactile myography* (TMG). The term TMG is used for high-density FMG: a technique in which many force/pressure sensors are put in contact with the subject's limbs. TMG has already been proved at least in Radmand et al. (2016) and Jaquier et al. (2017) and it has been shown to offer an unprecedented detail about the muscle patterns under examination. However, in these different works, combined motions were not tested. On another note, TMG can also be embedded in a socket-like structure or in a shape-conformable bracelet for ease of use and the bare application of linear regression on its values yields good results in intent detection.

Specifically, we hereby show that TMG and linear regression can be used proficiently in an online goal-reaching task, and that it suffices to gather data from the subject for single actions only (i.e., a list primary actions that we define in Subsection 3.4) to also be able to predict combined actions (e.g., flexing and pronating the wrist at the same time). Firstly, we carry out a study of existing literature about *muscle activation limits* in complex actions, and propose a set of physiologically feasible maximal activations, apt for any future experiment involving combined actions. Indeed the physiology of the hand and wrist as well as forearm limits the possibilities of mobilizing multiple muscles at the same time. For instance, each forearm or wrist action has an influence on the level of power grasp's strength one is able to produce.

Furthermore, we engage 12 subjects and one amputee in an instance of the Target Achievement Control (TAC) test (Simon et al., 2011) with control over 3 DoF^3^. Our experimental results show that, when using linear regression and TMG, combined actions can be predicted by gathering data about single movements only. However, power grasping seems to have a remarkable negative influence on the test. The high resolution of TMG is probably the reason why linear regression suffices. Actually, in other cases in which no high-resolution approach could be used, researchers needed to resort to artificial combinations of existing data clusters (Nowak and Castellini, 2015, 2016; Nowak et al., 2016), especially when dealing with more than 2 DOFs. In these works we have already proposed to “dope” the dataset of a myocontrol system with synthetic data obtained by linearly combining pre-existing sEMG patterns, in order to be able to predict combined activations of complex actions without the need to gather data directly related to them. In this study, too, we compare using bare linear regression on the sensor values with a set of features reducing the dimensions of the input space to one sixth. This idea could help whenever limited computational power is available, e.g., in a future implementation running on an embedded battery-powered prosthesis controller.

###  Related Work

Previous research proposed control over combined actions with electromyography. In particular, Jiang et al. (2009) did an offline analysis of EMG data collected with restriction of the wrist and proposes a linear model (non-negative matrix factorization - NMF) built on neural muscle synergies: single-DoF wrist activations are extracted by a linear decomposition of the sEMG signals.

Several studies also used this idea of a linear decomposition of the sEMG signals. For example, Nagata and Magatani (2011) presents a preliminary offline experiment where the high-density electromyography (HD-EMG) data of only two subjects performing combined motions is collected and activations are separated by a Canonical Discriminant Analysis to construct basic motions. Furthermore, in Yatsenko et al. (2007), a PCA-based (Principal Component Analysis) technique built on sEMG energies is used offline to separate combined hand and wrist motions. Despite the limited number of subjects (2 intact subjects and 1 amputee), they present preliminary results of the same combined motions presented in our experiment, i.e., wrist flexion/extension, wrist pronation/supination, and power grasp.

In Kent and Engeberg (2011), control of the combined 2 DoFs power grasp and wrist flexion/extension is possible thanks to a biomimetic controller taking into consideration the muscular structure of the forearm. Amsuss et al. (2014) tested the same NMF algorithm with muscle synergy-inspired decomposition as in Jiang et al. (2009), combined with a Linear Discriminant Analysis, in a free test of 1h in which 2 amputees tried rotation of the wrist combined with wrist flexion/extension while manipulating objects and performing a clothes pin test. The subjects were equipped with a socket containing 8 EMG Ottobock electrodes. Unfortunately, training the NMF algorithm with the same 3 DoFs, as in our study, resulted in very unreliable results. Using only 8 EMG electrodes and simple linear regression, Hahne et al. (2018) tested the control of combined wrist rotation and grasping on 5 amputees in a series of activities of daily living. In this paper, they used a control scheme based on non-intuitive mapping. It consists in a training phase based on motor skill learning and brain plasticity, i.e., the subject is involved in a longer signal-inspection phase where the experimenter searches for the best-looking signals and uses the associated movements to train the algorithm: for example, a radial/ulnar deviation can be mapped to a power grasp. The cognitive load of such a training is thus higher than in the case of a direct mapping like in our case. Additionally, it limits the number of DoFs that can be controlled simultaneously.

HD-EMG was also investigated for combined motions in several articles. For example, Ison et al. (2016) applied motor skill learning, a.k.a. non-intuitive mapping, and HD-EMG in an online experiment to control a 7-DoF robotic arm. In their experiment, it was possible for the subjects to switch in between 2 modes of each 4 DoFs (with a common DoF between the 2 modes being the power grasp); meaning simultaneous control could be established over 4 DoFs. Finally, Muceli et al. (2014) realized a similar online experiment as the one presented in our research and showed that, using reduced HD-EMG, with 2 DoF fed to the NMF machine learning, the subjects could control combined actions.

## 2. Limitation of the Power Grasp Strength With Relation to the Hand and Wrist Biomechanics

Although the functional Range of Motion (ROM) of the hand and wrist joints has been studied in several papers (Palmer et al., 1985; Hume et al., 1990; Ryu et al., 1991), while combining hand and wrist movements, limitations come into place. Indeed, wrist and hand movements are due to combinations of muscle synergies (Mussa-Ivaldi et al., 1994; D'Avella et al., 2003); changing the forearm position can potentially change the length of the extrinsic muscles of the hand, which determine most of the grip strength. The combinations of joints' ROM can thus be altered (Brand and Hollister, 1999). This point needed to be taken into account in order to provide realistic and feasible targets for hand/wrist positions to our subjects. For this reason, in the following section we study the limits of the hand and wrist's joint motions.

Several studies have shown that forearm and wrist positions have an influence on power grasp (Terrell and Purswell, 1976; Richards et al., 1996; Claudon, 1998; De Smet et al., 1998; Mogk and Keir, 2003) or other types of grasps (Dempsey and Ayoub, 1996). The shoulder position also has its influence (Halpern and Fernandez, 1996; Kattel et al., 1996). Both the physiological cross-sectional area (PCSA) and the length-tension relationship of a muscle have their influence on determining to which extent they contribute to one action (Zellers and Hallbeck, 1995; Brand and Hollister, 1999). In order to produce maximal contraction, each muscle has its optimal length, any elongation or shortening of the muscles dedicated to the finger and thumb flexion could have an influence on the power grasp strength (Brand and Hollister, 1999). Changing the configuration of the arm (at the shoulder, elbow, or wrist joints) physically affects the spatial relationship between the extrinsic muscles of the hand and wrist.

*Wrist flexion/extension and power grasp:* Studies have shown that the maximum voluntary contraction (MVC) of the hand decreases with wrist flexion and increases with wrist extension (Claudon, 1998; Fong and Ng, 2001; Bhardwaj et al., 2011), sometimes to a higher level than the one in neutral position (despite some studies showing the contrary Terrell and Purswell, 1976; Mogk and Keir, 2003, probably due to the angle of wrist extension). This phenomenon is actually an orthopedic observation known under the name of *tenodesis*. This can be a result of the long flexor and extensor muscles of the fingers passing through the wrist, finger and elbow joints: they work in synergy to stabilize the intermediate wrist joints and to activate the distal joints, such as the ones of the fingers (Richards et al., 1996). This synergy between the finger flexors and extensors allows, once the wrist joint is stabilized, an optimal flexion of the finger joints, i.e., a maximal power grasp strength (Austin, 2005). Moreover, a wrist extension brought by the ECU, ECRL and ECRB^4^ muscles generate a passive tension in the extrinsic finger tendons (FDS and FDP), which are stretched over the extended wrist. When considering this and the previously mentioned synergy to stabilize the wrist, one could conclude that the reciprocal relationship between the wrist muscles and the finger flexors is the reason for an optimal power grasp in a slightly extended and stable wrist position. On the contrary, during a wrist flexion, the tendons of the FDS and FDP release, while the tendons of the ED, EDM and EPL distend: this passive tension on the finger extensors allows the fingers to stretch. This has been confirmed in an electromyographic study by Claudon in which he shows that the activity of the ED is lower than that of the FDS during maximal extension of the wrist; he also observed the opposite result in maximal flexion (Claudon, 1998).

*Wrist flexion and thumb:* Additionally, as for the extrinsic muscles of the fingers, the wrist position also influences the thumb: during wrist flexion, the flexion at the interphalangeal joint of the thumb (due to the FPL) is significantly reduced (Austin, 2005); hence the power grasp is furthermore hindered.

*Wrist supination/pronation and power grasp:* Several researchers have also studied the influence of forearm rotation (pronation/supination) in relation to the grasp strength (Terrell and Purswell, 1976; Marley and Wehrman, 1992; Richards et al., 1996; Claudon, 1998; De Smet et al., 1998; Mogk and Keir, 2003). It has to be noted that though often referred as a DoF of the wrist joint in the robotic field, the forearm/wrist rotation is biomechanically an elbow DoF. Most of the studies show that a forearm pronation decreases the grip strength while a supination tends to increase it (Terrell and Purswell, 1976; Richards et al., 1996; Claudon, 1998; De Smet et al., 1998). It is supposed that the decreased strength in supination shown in two studies (Marley and Wehrman, 1992; Mogk and Keir, 2003) can be due to the method used or due to the angle of supination and pronation. One explanation to the increased strength during supination could be that in this position, the long flexors of the fingers (ED, FDP, FDS) are able to contract maximally. Indeed, to move the wrist from a supinated to a pronated position, the radius rotates over the ulna and the extrinsic flexor muscles of the fingers are wrapped around the radius during the rotation (Richards et al., 1996). This could result in a change of the length of these muscles, hence affecting their optimum length-tension relationship and reducing the strength of the power grasp.

A summary of the different studies analysing grip strength according to forearm and wrist motions can be found in Table 1. Not being used in our study, ulnar and radial deviations (Terrell and Purswell, 1976) were purposefully left out for the sake of readability of the table.

**Table 1:** Percentage of the maximal grip strength with combined wrist and forearm movements to estimate the thresholds for actions combined with a power grasp.

		**Pronation**			**Neutral**			**Supination**		
**Study**	***N*^k^**	**Extension (%)**	**Neutral (%)**	**Flexion (%)**	**Extension (%)**	**Neutral (%)**	**Flexion (%)**	**Extension (%)**	**Neutral (%)**	**Flexion (%)**
Bhardwaj et al. (2011)^a^	100				107	100	54			
Parvatikar and Mukkannavar (2009)^b^	50				91	100				
Mogk and Keir (2003)^c^	10	95	87	50	99	100	56	98	94	61
Fong and Ng (2001)	30				102	100				
Claudon (1998)^e^	15	93	84	62	104^d^	100	73	103	101	71
De Smet et al. (1998)^f^	40		92			100			101	
Kattel et al. (1996)^g^	15					100	73			
Richards et al. (1996)	106		91			100			102	
Zellers and Hallbeck (1995)	20				98	100	84			
Duque et al. (1995)^h^	20					100	52			
Marley and Wehrman (1992)^i^	20		80			100			90	
Terrell and Purswell (1976)^j^	40	69	88	57	77	99	70	77	100	73
**MEAN**		86	**87**	57	**97**	**100**	**66**	93	**98**	68

a *Force exerted while the wrist was immobilized at 30° flexion/extension*.

b *Based on reported grip strength for shoulder at 0° of shoulder flexion and elbow at 90°*.

c *Peak force was used to evaluate maximum grip strength 100% of MVC*.

d *Force exerted while the wrist was immobilized at 30° extension*.

e *Based on maximal voluntary flexion/extension*.

f *Non-immobilized wrist, full pronation/supination*.

g *Based on reported grip strength for shoulder at 0°, elbow at 90° for all actions and when wrist flexion is involved, it is with 2/3 of maximum flexion*.

h *Based on reported percentage in full voluntary flexion*.

i *Based on reported percentage at 90° of elbow flexion*.

j *Force exerted with the wrist at 50° of extension*.

k *Number of participants to the study*.

*The values in bold are the ones used as references for choosing the experiment's thresholds (cf. Table 2)*.

## 3. Materials and Methods

### 3.1. Experimental Setup

#### 3.1.1. Tactile Bracelet

**Figure 1:**
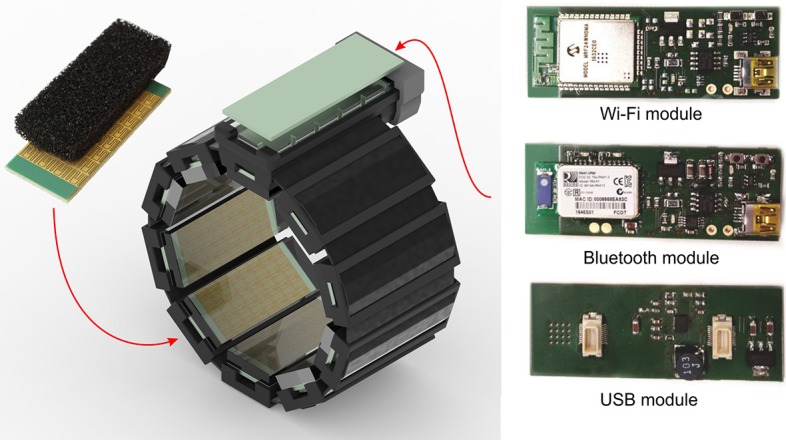
The second generation tactile bracelet: **(left)** the bracelet and a single sensor module in the upper left; **(right)** three communication modules — USB, Bluetooth and Wi-Fi. The wireless modules include the circuitry for battery charging, powered through a dedicated USB connection.

For the experiment presented in this work, we used an improved version of our previously developed tactile bracelet (Kõiva et al., 2015) [cf. Figure 1]. The tactile bracelet, worn typically around the forearm, measures the bulges of the muscles with a spatial resolution of 5 mm. Depending on the thickness of the arm, up to ten sensor modules, with 4 × 8 tactile cells each, can be used. The modular design and the attachment around the arm using hook-and-loop band allow the bracelet to conform on various arm sizes and shapes, the latter especially important in case of residual limbs. The data from up to 320 tactile cells is sampled at 100 Hz.

**Figure 2:**
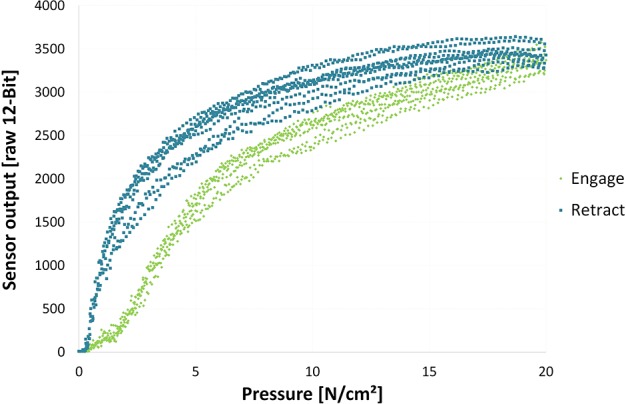
Sensor characteristics measured over 10 trials from no contact to 20 *N*/*cm*^2^ and back to no contact using the new 3 mm thick PANA Foamtec GmbH PE-K45EVAELS. The green samples are collected while pressure onto the sensor was increased whereas the blue ones are sampled during the retraction phase.

The improved second generation tactile bracelet has more robust sensors and improved readout electronics. The conductive elastomer foam located as the outmost surface of the sensors and the material touching the human skin, was changed to 3 mm thick PANA Foamtec GmbH PE-K45EVAELS, a closed-cell cross-linked polyethylene foam with EVA content. The more dense foam made the tactile bracelet significantly more robust and less tear-prone when accidental shear forces are exerted during handling, e.g., while donning and doffing it. Figure 2 shows the sensor characteristic using the new elastomer foam.

As is apparent from Figure 2, the taxel response is not linear. This helps to exploit the sensors' dynamic range as much as possible. We intentionally decided not to linearise the response in order to save computational effort, and not alter the signal-to-noise ratio. See, e.g., Castellini et al. (2018) for more thorough description of the device and its pros and cons.

**Figure 3:**
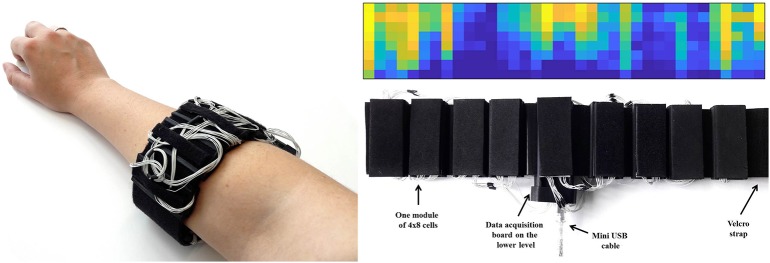
Picture of the tactile bracelet, consisting of 9 boards of 32 cells each (8 vertical and 4 horizontal), and visual representation of the data.

The readout electronics was completely redesigned to make use of a newer microcontroller model and freshly rewritten software stack. The second generation tactile bracelet readout electronics uses a Microchip PIC32MZ microcontroller, running at 200 MHz. The software was rewritten to make use of FreeRTOS real-time-operating-system, resulting in a greatly simplified firmware code while still maintaining precise timing required to read out the high number of AD7490 ADCs connected to high-impedance tactile cells. The bracelet can now optionally be used in battery-powered wireless mode, the captured signal being transmitted over Bluetooth or Wi-Fi. For the experiment in this paper though, the wired USB connectivity was used. A described picture of the tactile bracelet and a visual representation of the data can be found in Figure 3.

### 3.2. Data Processing and Intent Detection

#### 3.2.1. Machine Learning Algorithm

The algorithm of choice was ridge regression (Hoerl and Kennard, 1970), already used successfully several times with the tactile bracelet, in e.g., Nissler et al. (2017). The ridge regression algorithm estimates the parameters of a mapping matrix **W** ∈ ℝ^*D*×*d*^, with *D* the number of input sensors or features and *d* the number of output activations. We call it “activation” because it stems from the voluntary activation of a set of muscles, which we somehow recorded (i.e. wrist flexion and extension are two different activations). This mapping matrix **W** is based on a set of *m* training pairs of input-output values, **X** ∈ ℝ^*m*×*D*^ and **Y** ∈ ℝ^*m*×*d*^, and the regularization coefficient λ, uniformly set at the standard value of 1 in this case (also previously chosen in Nissler et al., 2017). We obtain the mapping matrix with the following equation:

(1) $$\begin{array}{l} \text{W}={\left({\text{X}}^{T}\text{X}+\lambda \text{I}\right)}^{-1}{\text{X}}^{T}\text{Y} \end{array}$$

The output vector $\hat{\text{y}}\in {\mathbb{R}}^{d}$, resulting from the input vector $\hat{\text{x}}\in {\mathbb{R}}^{D}$, is then equal to:

(2) $$\begin{array}{l} \hat{\text{y}}={\text{W}}^{T}\hat{\text{x}} \end{array}$$

The position of the virtual hand was then controlled thanks to this output vector, having one value between 0 and 1 for each activation.

#### 3.2.2. Feature Selection

Captured tactile data was filtered by a low-pass first-order Butterworth filter with a cut-off frequency of 1 Hz to attenuate the high-frequency noise, which was previously tested in an initial round of experiment and showed not to impair the speed. In this study, two feature selection methods were used in order to determine the prediction.

The first one had already been used successfully in previous online studies with the first generation tactile bracelet (Jaquier et al., 2017; Nissler et al., 2017) and consists of the unprocessed data (except the Butterworth filter mentioned above) directly fed to a simple ridge regression (RR) algorithm. More precisely, the data consists of 288 filtered sensor data (9 boards of the 32 sensors each).

The second feature selection method, Gradient-based features extracted from Regions of Interest (ROIs) (Haralick and Shapiro, 1992), has already been used in ultrasound image processing and more specifically to identify finger movements (Castellini et al., 2012; Sierra González and Castellini, 2013; Ortenzi et al., 2015), also together with regression-based algorithms (Castellini et al., 2012; Sierra González and Castellini, 2013). More recently, this method has been further tested in an offline study investigating different methods of feature extraction for the Tactile Bracelet: the ROI gradients gave the highest classification accuracy (Castellini et al., 2018) over Harris corner extraction (Harris et al., 1988) and the structural similarity index (Boschmann and Platzner, 2014) on bicubic interpolated data. Unlike the round-shaped overlapping ROIs used in Sierra González and Castellini (2013) for ultrasound image processing and due to the low resolution of the tactile bracelet compared to ultrasound, a simpler strategy was adopted here after several pre-tests, delimiting each ROI as a non-overlapping 4 × 4 taxel square (Castellini et al., 2018) (a taxel being the value of one sensor), resulting in two ROIs per board (cf. **Figure 5**). Three features (α_*i*_, β_*i*_, γ_*i*_) were extracted from each *ROI*_*i*_, with *i* ∈ [[1, 18]]. These features can also be considered as a vector that represents the second moment axis of the ROI area, i.e., the line around which the ROI would have the lowest moment of rotation if it were cut from a rigid and uniform cardboard (Russ, 1999) or the normal line to the planes that best fits all the observed taxels of the ROI. The value distribution of the ROI is approximated by a first order regression plane:

(3) $$\begin{array}{l} \hat{G}\left(x,y\right)=\alpha_{i}\left(x-x_{i}\right)+\beta_{i}\left(y-y_{i}\right)+\gamma_{i}. \end{array}$$

where Ĝ(*x, y*) is the point on the fitted plane at the position (*x, y*), and (*x*_*i*_, *y*_*i*_) the interest point defined in the upper left corner of the *ROI*_*i*_ (Castellini et al., 2018).

The least squares fit to the observed gray values *G*(*x, y*) of the ROI and is obtained from α_*i*_, β_*i*_, and γ_*i*_ that minimize the sum of the squares of the distances between our points and the plane:

(4) $$\begin{array}{l} \varepsilon^{2}=\underset{\left(x,y\right)\in ROI_{i}}{\sum }{\left[\alpha_{i}\left(x-x_{i}\right)+\beta_{i}\left(y-y_{i}\right)+\gamma_{i}-G\left(x,y\right)\right]}^{2}. \end{array}$$

**Figure 4:**
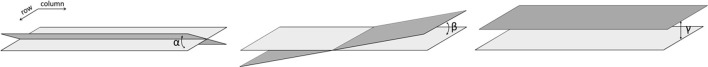
Visualization of the parameters alpha, beta, and gamma of a plane.

Represented in Figure 4 for one ROI only, α denotes the mean image gradient along the x direction (row), β along the y (column) and γ is an offset (mean gray value of the taxels in the ROI after resolution of the equation).

The computation of the parameters for one ROI is performed with ridge regression:

(5) $$\begin{array}{l} \;w={\left(A^{T}A+\lambda I\right)}^{-1}A^{T}r,\\ \text{with }w=\left[\begin{array}{c} \alpha_{i}\\ \beta_{i}\\ \gamma_{i} \end{array}\right],A=\left[\begin{array}{ccc} 1 & x_{1} & y_{1}\\ 1 & x_{2} & y_{2}\\ \vdots & \vdots & \vdots \\ 1 & x_{l} & y_{l} \end{array}\right],\text{and }r=\left[\begin{array}{c} ROI_{i}\left(x_{1},y_{1}\right)\\ \vdots \\ ROI_{i}\left(x_{l},y_{l}\right) \end{array}\right]. \end{array}$$

Solution of the linear regression, **w** contains the parameters α_*i*_, β_*i*_, and γ_*i*_, while *A* contains the coordinates of the ROI and **r** contains the gray values of the ROI in an *l* × *l* matrix, with *l* being the side length of the square ROI.

**Figure 5:**
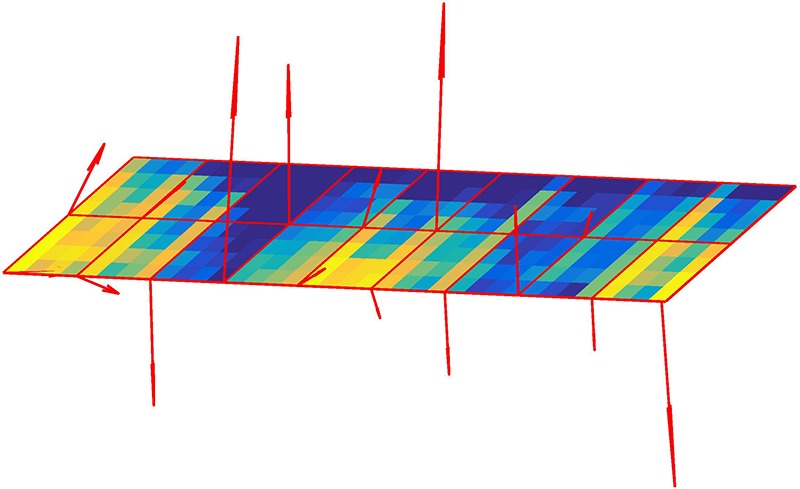
A schematic representation of ROIs and their gradient, obtained from real data.

A representation of the ROIs and the gradients can be seen in Figure 5. Since we had 2 ROIs on each of the 9 sensor boards and that three features were extracted from each ROI, a 54-dimensional feature vector was fed to the RR algorithm, namely RR-ROIG in this article.

### 3.3. Participants

Twelve able-bodied right-handed volunteers participated in the experiment (30.6 ± 6.6 years old, three females and nine males). One left-hand amputee (male, 35 years old) also took part in the experiment. He was trans-radially amputated in 2005 and uses daily, since 2012, a Variplus prosthesis by Ottobock with standard two-electrode control and no rotation unit on the device.

The experimental procedure was thoroughly explained to the participants in both oral and written form before the experiment and each of the participants was given a written informed consent form. The experiment was performed according to the WMA Declaration of Helsinki and was preliminarily approved by the Work Ethical Committee of DLR.

### 3.4. Experimental Protocol

**Figure 6:**
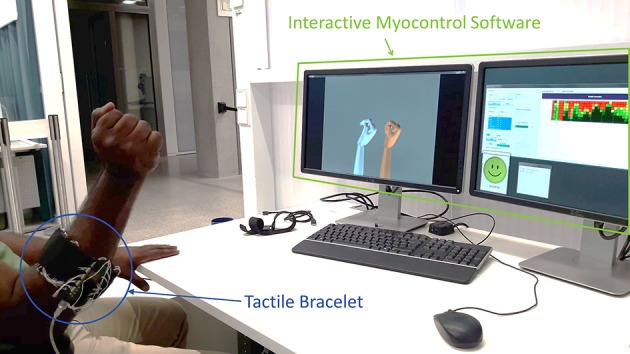
A bird's-eye view of the experiment.

Each subject would sit comfortably, their back against the backrest of the chair and their elbow placed near the furthest edge of the armrest relative to them (cf. Figure 6). The hand with which the participants would perform the experiment would be alternatively switched between subjects. The tactile bracelet was placed at the lower arm location with the greatest muscle bulge, i.e., near the proximal end of the forearm, as shown in Figure 6. In order to avoid any undesired change in the signals due to tissue accumulation between the tactile bracelet and the inner side of the elbow, the subjects were told to keep their forearm at an angle of 90° with their upper arm. The default/resting hand position was a flat hand with the palm facing the side of the body.

The experiment consisted of a training session on a set of single activations of the hand and wrist actions (*rest, power, flexion, extension, pronation, supination*) and of tasks to reproduce single and combined actions that are detailed in Table 2. The above mentioned set of actions (except “rest”) are labeled as “single actions”: subjects train on them and these actions should be reproduced individually during the task reaching test. Additionally, we define any combination of single actions as combined actions. Each action to be reproduced had a specific level to make it realistically possible in terms of muscle coordination and to prevent muscle overload (cf. Section 2).

**Table 2:**
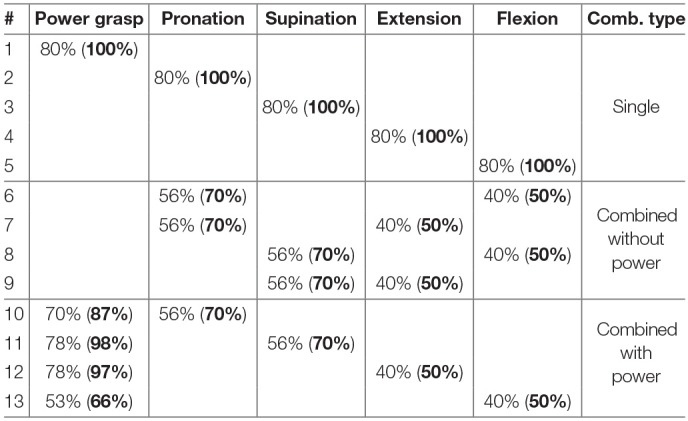
Single- and multi-action combinations performed during the experiment with the different thresholds chosen for each action with an 80% factor.

*The values in bold represent the ones extracted from the literature or chosen from a pre-round of testing in the case where no power grasp was involved*.

In order to set the different levels and limitations of the power grasp for the visual stimulus, an average over all studies (over the methods) of Table 1 was calculated.

Since to the best of our knowledge there are no studies on the limitation of forearm and wrist DoFs without the power grasp (i.e., pronation/supination and flexion/extension), we defined these specific threshold limitations according to an initial round of trials. The amputee performed the experiment with the same target thresholds as the able-bodied subjects.

We added an alleviating factor of 80% to these extreme limits of the hand and wrist movements in order to make the experiment more comfortable for the participants and to leave a safety margin to impossible target positions.

On the computer screen, positioned in front of the participants, two realistic 3D hand models were displayed: one acted as the visual stimulus, which pose the subject had to match with his/her own hand (or with a virtual hand prosthesis in case of the amputee) and the second one showed the predicted intended movement, calculated by the machine learning algorithm working on the captured tactile bracelet data. The experiment started with a training phase, in which the recording would take around 2 min: each participant was asked to perform three repetitions of the stimulated single actions defined previously. The data collected during this phase was used to train the learning machines (RR and RR-ROIG as explained in Subsection 3.2), which in the later stage of the experiment would drive the second hand model. Then, in order to counter the learning effect and to adapt to the system, each participant was asked to perform a familiarization phase, consisting of six tasks to reproduce. Additionally, pressure-based data are prone to drifting: a problem that was already identified in Castellini et al. (2018). This is supposedly due to the elasticity of the skin and the memory effect of the foam (cf. Subsection 3.1.1). To avoid this issue, 2 repetitions of training were inserted after the familiarization phase and in between each repetition of task-reaching phases.

**Figure 7:**
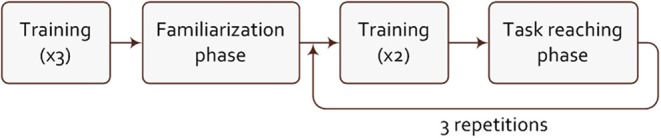
Diagram of the experimental procedure.

A task-reaching phase is a TAC test consisting of 26 tasks (13 tasks for each tested machine learning algorithm) in a randomized order while still maintaining an alternation between RR and RR-ROIG, every other subject starting with RR. These tasks can be single actions, or a simultaneous combination of actions with a certain percentage of the full activation defined in Table 2. The desired task was demonstrated by the visual stimulus and the subject had 15s to match it with the second virtual hand, driven by his muscle stimulus. To succeed in the task, the participant had to keep the controlled virtual hand in the same position as the visual stimulus for 1.5 s. Matching was defined as remaining within 20% of each target activation value. For the amputee this error threshold was set to 25% and the starting machine learning was RR. The task-reaching phase was repeated 3 times with 2 repetitions of training in between each of them in order to counter the drift induced by the bracelet. Figure 7 shows the experimental procedure.

To evaluate the performance of the participants for each action and the different machine learning algorithms, we assessed the success rate over all 39 tasks (SR - Success Rate), the time that the subject took to accomplish the task (TCT - Time to Complete Task) and the cumulative time in the target (TIT - Time in Target). A video of the TAC test showing the experiment with an amputee and one able-bodied participant can be found in the Supplementary Material.

### 3.5. Statistical Analysis

For the statistical analysis we used the libraries provided by the programming language R. We first performed a Friedman test to see the influence of the repetitions on the SRs, which could eventually indicate a learning effect. Its result (*p* = 0.3927) showed that there was no statistical difference between the three repetitions of the TAC test. We can suppose that the familiarization phase was efficient and we will therefore aggregate the data of the 3 repetitions for the rest of the analysis. To evaluate the difference of SRs between the two feature selection methods, we tested the normality of the aggregated dataset with both Shapiro–Wilk and Jarque–Bera normality tests which both concluded that the distribution of the data is not significantly different from normal distribution. Therefore, we performed a paired *t*-test on the two feature selection methods. Then, after an initial round of analysis between “single actions” and “combined actions,” we realized that the power grasp was creating the difference between the two groups. For this reason, we decided to carry out the analysis with the Holm-Bonferroni adjustment method for these three subgroups: “single actions,” “combined actions without the power grasp,” and “combined actions including the power grasp.” We tested the normality of the aggregated datasets before each pairwise statistical test with both Shapiro–Wilk and Jarque–Bera normality tests. Both tests resulted in not rejecting the normality hypothesis. To assess the difference between the three previously mentioned subgroups, we used the multiple pairwise *t*-test with Holm-Bonferroni adjustment method as well as Cohen's *d* effect size. Regarding the time-related performance measures, no inferential statistical test was performed due to the fact that the condition of completeness of the dataset was not observed: the TCT can only be considered for successful tasks since a time limit of 15 s was fixed and, for the same reason, we decided to separate the analysis of the TIT between successful and failed tasks. Therefore, we kept this part of the statistical analysis on a descriptive level.

## 4. Experimental Results

This section provides the results of the experiment described in the previous section. First, a detailed analysis on the results of the 12 able-bodied participants will be presented. In particular, in Subsection 4.1, we compare the two machine learning methods, and present the difference between the single and the combined actions and the success rate action by action. In Subsection 4.2, we analyse the results of the TCT and TIT relative to the learning algorithm and the type of combined movement. Then, we analyse the results of the amputated subject in Subsection 4.3.

### 4.1. Success Rate

**Figure 8:**
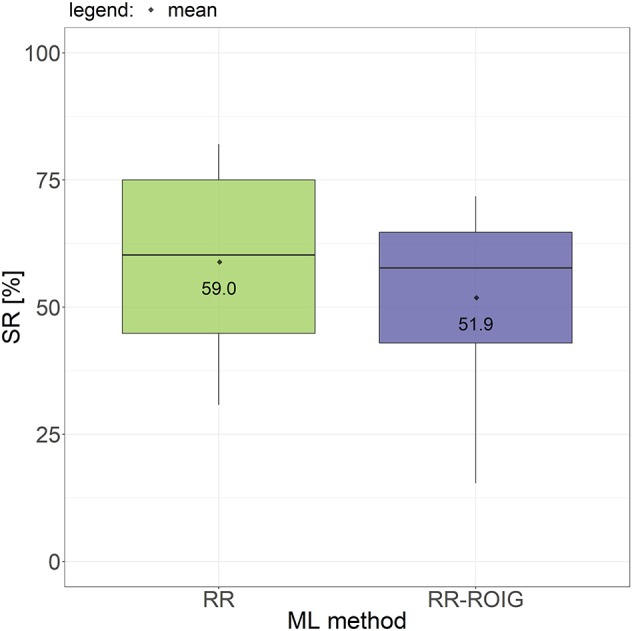
Boxplots and means of the success rates for all tasks across all participants grouped according to the machine learning method used.

Figure 8 shows the difference of the success rates according to the machine learning method tested across all subjects. No statistically significant difference can be observed (paired t-test *p* = 0.2123), although the average performance of RR was around 8% better than that of RR-ROIG (59.0 ± 17.6% vs. 51.9 ± 17.2%).

**Figure 9:**
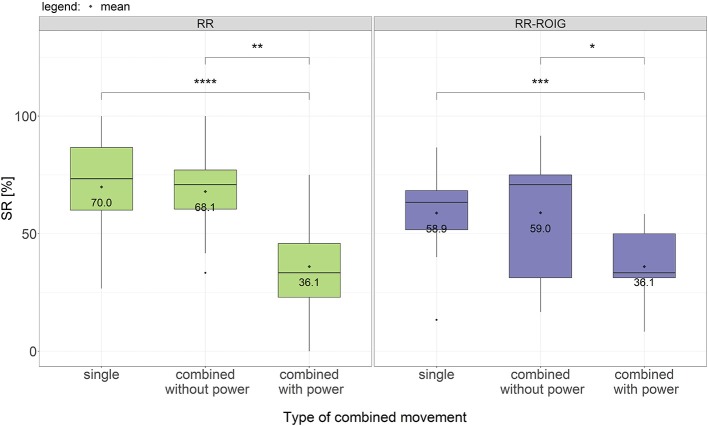
Boxplots and means of the success rates across all participants grouped according to the machine learning method and the type of combined movement used. *p ≤ 0.05; **p ≤ 0.01; ***p ≤ 0.001; ****p ≤ 0.0001.

When comparing the success rate action-wise, we can subdivide it in three groups: “single actions,” “combined actions without the power grasp,” and “combined actions including the power grasp” (as detailed in Table 2). The SR of RR and RR-ROIG in each of these three groups is described in Figure 9. The difference within each group is also not statistically significant between the two algorithms: *p* = 0.17 for “single actions,” *p* = 0.48 for “combined actions without the power grasp” and *p* = 0.86 for “combined actions including the power grasp.”

As we can take from Figure 9, the average SR of the “combined actions including the power grasp” — for RR 36.1 ± 21.7% — is much lower, by more than 30%, than the average SRs of the two other groups — for RR 68.1 ± 20.4% for “combined actions without the power grasp” and 70 ± 21.2% for “single actions.” This difference is also present for the RR-ROIG algorithm with 22.8% between the SR of “combined actions without the power grasp” and the SR of the two other groups. In order to assess the significance of this difference, a multiple paired sample *t*-test with Holm-Bonferroni adjustment method was performed.

For RR, the difference between “combined actions including the power grasp” is highly significant when compared with “single actions” (*p* = 0.0001, *d* = 1.5802) as well as when compared with “combined actions without the power grasp” (*p* = 0.0039, *d* = 1.5178) after the Holm–Bonferroni correction. For RR-ROIG, the difference between “combined actions including the power grasp” is significant (*p* = 0.0007, *d* = 1.3362) when compared with “single actions,” and also significant when compared with “combined actions without the power grasp” (*p* = 0.0310, *d* = 1.0764) after the Holm–Bonferroni correction. For both methods, there is no significant difference between “single actions” and “combined actions without the power grasp.”

**Figure 10:**
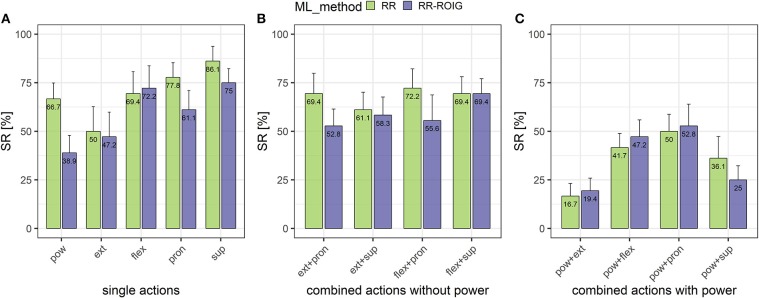
Means and standard errors of the success rates per actions separated into 3 groups: single actions **(A)**, combined actions without the power grasp **(B)** and combined actions including the power grasp **(C)**.

Figure 10 describes the SR action-wise among all participants. The “combined actions including the power grasp” are seemingly lower than the “single actions” and the “combined actions without the power grasp.” In particular, power grasp combined with wrist extension and power grasp combined with supination seemed to be the most difficult tasks to achieve.

### 4.2. Time-Related Metrics

Other than the success rate, the Time to Complete the Task (TCT) and the total cumulative Time In the Target (TIT) were also measured.

**Figure 11:**
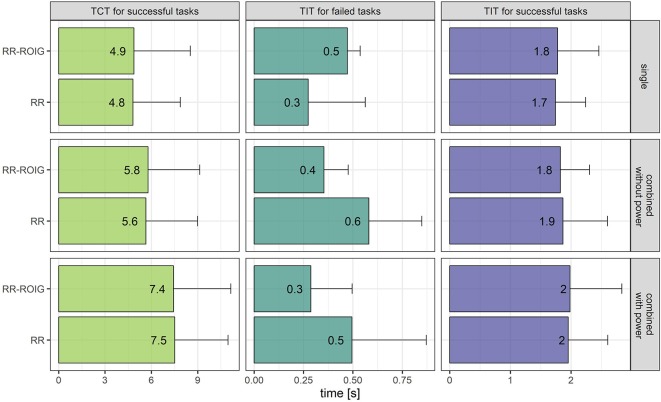
Means and standard deviations of the TCT for successful tasks, TIT for failed tasks, TIT for successful tasks according to the machine learning method and the type of combined movement.

*TCT for successful tasks:* We can see in Figure 11 that the TCT for “single actions” and “combined actions without the power grasp” was almost 2 s faster than the TCT for “combined actions including the power grasp,” which would indicate that actions combined with a power grasp were harder to reach.

*TIT for failed tasks:* For the combined actions in general, irrespective if the power grasp was included or not, the TIT was higher with RR than with RR-ROIG. On the contrary for single actions, the TIT seems higher with RR-ROIG. For all failed tasks the TIT is relatively low compared to the fixed goal of 1.5 s.

*TIT for successful tasks:* The cumulative TIT for successful tasks is relatively close to the targeted time of consecutive 1.5 s: this means that when a task could be completed, it would usually be achieved without wobbling around the goal and it would not depend on the type of action it is (combined or not).

### 4.3. Results Obtained by the Amputated Subject

The amputee achieved 20.5 ± 21.7% of success rate over all tasks with RR-ROIG and 15.4 ± 25.9% with RR. We here introduce a new performance index of reachability to verify if the task was reached by being in the target for one sample) at least once during the TAC test. The SR is however still measured the same way as previously described in the Experimental Procedure section, the subject having to keep the target during 1.5 s within the time of 15 s. As shown in Table 3, half or more of the types of action were attainable at least once during the three repetitions for each of the three combination groups. However, the success rates were on average 17.9 ± 23.5%. By calculating the time to complete the successful tasks, Table 3 shows that it took on average 5.1 ± 4.3*s* for “single actions,” 7.6 ± 4.1*s* for “combined actions without the power grasp,” and 9.0 ± 5.0*s* for “combined actions including the power grasp.”

**Table 3:** Means and standard deviations of the reachability of the tasks, the success rate, the time in task and the time to complete the successful tasks for the amputee according to the type of combined movement.

**Type of combined movement**	**ML method**	**Reachable (%)**		**SR (%)**		**TIT (s) [successful tasks only]**		**TCT (s) [successful tasks only]**	
Single	RR	60.0 (54.8)	50.0 (53.5)	13.3 (18.3)	13.3 (17.2)	2.1 (0.8)	1.8 (0.6)	7.0 (6.2)	5.1 (4.3)
	RR-ROIG	40.0 (54.8)		13.3 (18.3)		1.5 (0.0)		3.2 (1.6)	
Combined without power	RR	25.0 (50.0)	62.5 (51.8)	16.7 (33.3)	25.0 (29.5)	3.1 (0.2)	2.3 (0.8)	9.6 (3.2)	7.6 (4.1)
	RR-ROIG	100.0 (0.0)		33.3 (27.2)		1.8 (0.6)		6.6 (4.5)	
Combined with power	RR	25.0 (50.0)	50.0 (52.7)	16.7 (33.3)	16.7 (52.2)	1.9 (0.5)	1.7 (0.3)	7.3 (7.8)	9.0 (5.0)
	RR-ROIG	75.0 (50.0)		16.7 (19.2)		1.5 (0.0)		10.6 (1.7)	
All actions	RR	38.5 (50.6)	53.8 (50.8)	15.4 (25.9)	17.9 (23.5)	2.4 (0.8)	2.0 (0.7)	8.0 (1.8)	7.3 (4.3)
	RR-ROIG	69.2 (48.0)		20.5 (21.7)		1.7 (0.4)		6.8 (4.2)	

When comparing the same data grouped according to the repetition of the task-reaching phase in Table 4, we can see that in the first repetition the participant achieved relatively high reachability with an average of 38.5 ± 49.6% and a success rate of 46.2 ± 51.9% for the RR-ROIG algorithm. The two remaining repetitions did not seem to increase the SR nor the reachability of the tasks with respectively 15.4 ± 36.8% and 26.9 ± 45.2% for repetitions 2 and 3.

**Table 4:** Means and standard deviations of the reachability of the tasks, the success rate, the time in task and the time to complete the successful tasks for the amputated subject according to the three repetitions of the task-reaching phase.

**Repetition**	**ML method**	**Reachable (%)**		**SR (%)**		**TIT (s) [successful tasks only]**		**TCT (s) [successful tasks only]**	
Rep. 1	RR	23.1 (43.9)	38.5 (49.6)	15.4 (37.6)	30.8 (47.1)	3.0 (0.5)	2.0 (0.7)	10.1 (3.8)	6.9 (4.4)
	RR-ROIG	53.8 (51.9)		46.2 (51.9)		1.7 (0.5)		5.9 (4.3)	
Rep. 2	RR	7.7 (27.7)	15.4 (36.8)	7.7 (27.7)	7.7 (27.2)	1.5 (NA)	1.5 (0.0)	1.8 (NA)	4.3 (3.6)
	RR-ROIG	23.1 (43.9)		7.7 (27.7)		1.5 (NA)		6.9 (NA)	
Rep. 3	RR	30.8 (48.0)	26.9 (45.2)	23.1 (43.9)	15.4 (36.8)	2.2 (0.8)	2.1 (0.7)	8.6 (5.2)	9.4 (4.5)
	RR-ROIG	23.1 (43.9)		7.7 (27.7)		1.5 (NA)		11.9 (NA)	

Moreover, it has to be noted that for the second repetition of task-reaching phases, the control of the 3D-hand model was blocked after approximately half of the tasks and that the extension and power were problematic during the third repetition. This might be due to a drift in the signals or a sliding of the tactile bracelet on the stump that has a conical shape to which the tactile bracelet is not adapted.

**Figure 12:**
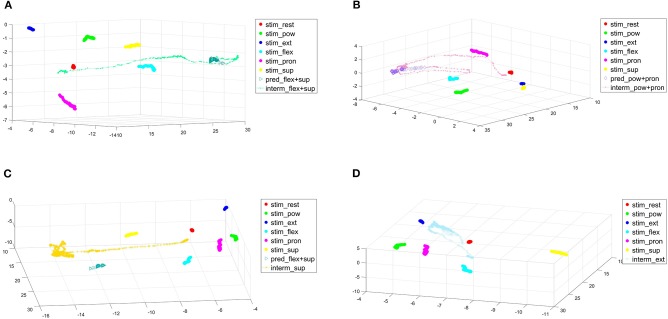
PCA of some actions (failed or non-reachable) performed by the amputee during the first repetition. Legend items starting with “stim,” “pred,” and “interm” represent respectively the trained action clusters, the samples when the subject is in the target and the intermediate values while trying to reach the target. **(A)** PCA with emphasis on failed flex.+sup. for RR, **(B)** PCA with emphasis on failed pow.+pron. for RR-ROIG, **(C)** PCA with emphasis on non-reachable supination action for RR, and **(D)** PCA with emphasis on non-reachable extension action for RR.

Additionally, some trajectories of reached-but-failed tasks and of non-reachable tasks are depicted in Figure 12 with PCA (Principal Component Analysis) for a 3D visualization with a percentage of variance of 88.41% for RR and of 84.82% for RR-ROIG. It can be seen in Figure 12 that the amputated subject went from the rest position (red) to a target position that seems to be a somehow linear combination of supination (yellow) and flexion (cyan). The subject then reached the target but did not successfully maintain the virtual hand in the position for the required amount of time of 1.5 s. Subjects were advised at the beginning of the experiment that it can be easier to first execute one of the actions of the combination and then the other one to move toward the target; however, they could choose which strategy to actually use. This proposed strategy can be very well seen in the top right subfigure, we can see that the subject first moved from rest to pronation to then additionally perform a flexion. The subject then reached the target for some time but did not manage to stay in the target for the required amount of time. The subject returned then slightly toward the rest position before trying again (which was also an advised strategy) and reaching the target for new samples, unfortunately not enough to achieve a successful task. The bottom left subfigure shows the subject trying to reach 80% of supination but going too far after the target and never reaching it. We can suppose that he has put too much strength into his muscles while trying to reach the target compared to the strength level he had performed during the training phase. The last subfigure (d) shows the trajectory going in the direction of the wrist extension cluster but slightly off, never reaching the 80% target. This shows, in our opinion, that the subject was able to control the hand but, due to different limitations (hardware, muscle fatigue, or simply not being able to reproduce the trained actions), was not able to perform well into our TAC test with the parameters and timings that we had set (15 s to achieve 1.5 s in task).

To get a better insight, a movie available in the Supplementary Material is showing part of the TAC test for the amputated subject.

## 5. Discussion

In this work we have first thoroughly examined the literature about the physiological limits of the hand/wrist complex, in particular, as far as the maximal combined activations of hand and wrist actions are concerned. This has allowed us to define a set of muscle activation limits for combined actions, which can, and to some extent, we claim, should be reused in similar experiments.

Of course, each amputation results in a different stump with a different muscle remnant configuration, and, to the best of our knowledge, biomechanical relationships among muscles in a stump cannot be estimated. But these findings could be taken as upper limits while designing a TAC test for amputees, since if an intact subject cannot reach a specific activation, reasonably, no amputee will be able to as well. Moreover, amputees usually strain their muscle remnants while activating them, since they lack proprioceptive feedback. Limiting the visual appearance of TAC test targets can only be beneficial.

Having determined this set of limits, we have then engaged a few intact subjects and an amputated person in such a test, aimed at checking how well TMG could be used to detect single and combined hand/wrist actions. The experimental results presented in the previous section indicate that TMG is viable for myocontrol, as it had already been discovered (see, e.g., Radmand et al., 2016; Jaquier et al., 2017; Nissler et al., 2017): The SRs obtained by our subjects are in line with these previous works.

But furthermore, and this may be the main finding of this work, the results denote that, by gathering data while the subjects perform single actions, TMG is able to predict *combinations of them*. This is of valuable interest, considering that it confirms previous results with HD-EMG (Muceli et al., 2014; Ison et al., 2016) and, as far as we know, it had not yet been discovered using TMG. We can suppose that, a high number of sensors, independent of the sensor type (force or sEMG), gives automatically the combined actions, provided a linear algorithm is used. TMG could detect single *and* combined actions, *with no statistically significant difference in the related success rates*. Notice that the SRs over all actions is slightly lower than the ones found in previous studies with TMG: 59.0% for RR in Figure 8 vs. 75.6% in Nissler et al. (2017) where no combined motions were tested. However, when we remove the “combined actions including the power grasp” of the picture, results average 69.1% of SR and are in line with previous literature for TAC tests. The action of power-grasping leads to a substantial reduction of the SR when combined with wrist movements. Although still more than one third of the tasks were achieved, this is a problem which needs to be tackled. In the current version of the bracelet, it is due to saturation of most of the taxels, in turn due to the large number of muscles involved in this action. (When combined with other actions, the problem is obviously amplified.) To solve this issue, further versions of the semi-conductive foam and tuning of the pull-up resistors of the controlling boards are being tested.

Although standard metrics were used, a comparison with other studies involving combined motions is difficult considering the different experimental conditions. Considering that offline and online analysis are hardly comparable, we will here only examine our results in contrast with articles including an online test. Muceli et al. (2014) present a very similar work with an online TAC test investigating the control of the 2 wrist DoFs. They show that a reduced version of HD-EMG (16 electrodes) fed to an NMF algorithm results in a successful prediction of combined movements. The tasks are however slightly easier to reach than ours: the required consecutive time in task is 300 ms (while the subjects had to maintain 5 times this duration in our experiment) and the time given to complete the task was 20 s (15 s in our case). In addition, in our experiment, subjects had a simultaneous control over 3 DoFs: the machine learning algorithm was fed with one additional pattern for the power grasp, which can interfere with other actions, and in particular wrist flexion that involves common muscles. Nonetheless, after recalculating our results with 300 ms of required consecutive TIT, we found an increase of more than 10% in the SRs (RR from 58.3 to 68.5% and RR-ROIG from 51.13 to 62.13%), while the TCTs decreased in general of 1.5 s or more and of 2 s in case of “combined actions including the power grasp.” Ison et al. (2016) accomplish control over 4 DoFs simultaneously where the participants had to control a 7-DoFs robotic arm in tasks such as grasping a tennis-sized ball and customized clothespins. However, the subjects were free in the sequence or combination of gestures to achieve the tasks. It needs to be noted that all of these articles use a non-intuitive mapping. Therefore, in these studies, the cognitive load of the training phase is supposedly higher than with the direct mapping that we use and the number of simultaneously controlled DoFs is limited in case no advanced surgical technique such as e.g., Targeted Muscle Reinnervation (Farina et al., 2017) is used. Another impressive work, also using non-intuitive mapping, is the one of Hahne et al. (2018), in which they focus on wrist rotation and grasping with 8 EMG and linear regression by involving 5 amputees in a series of prosthesis tests (clothespins and box and blocks tests). It is still a goal that has not yet been achieved by TMG and a limitation of our work, considering we only tested one amputee. However, TMG requires much less electronics for the same resolution, provides more stable signals (Connan et al., 2016) : Indeed force myography signals provide a stable plateau of activation while EMG signals present a peak of activation that decreases over time due to muscular motor-unit recruitment (Merletti et al., 2010a,b). Add in the fact that it is wearable, it could to a certain degree be easily integrated in a prosthesis. The computational burden required to extract the ROIG features is negligible, as already proved in (Sierra González and Castellini, 2013).

**Figure 13:**
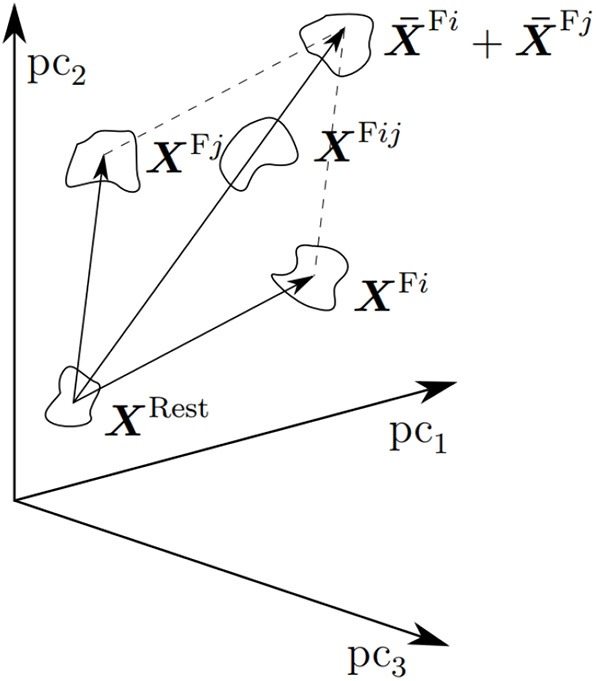
A graphical representation of linear superposition of effects in the input space. A rest cluster *X*^*Rest*^, two single-action clusters $X^{F_{i}}$ and $X^{F_{j}}$ and the combination cluster $X^{F_{ij}}$ are depicted. That linear regression can predict combined actions from single actions only, should imply that $X^{F_{ij}}$ largely coincides with $X^{F_{i}}+X^{F_{j}}$ (Reproduced with permission from Nowak, 2014).

That combined actions can be correctly detected using single-action data and linear regression indicates that linear superposition of effects might be present in the input space, as already suggested in Muceli et al. (2014). This fact needs be investigated in the future; it is likely that clusters of combined actions $X^{F_{ij}}$ should be to a large extent similar to the linear sum of the single-action clusters $X^{F_{i}}$ and $X^{F_{j}}$ they are composed of. A graphical representation of this issue is shown in Figure 13.

In this work, we also compared RR with RR-ROIG and showed in Figure 8 that the success rate of RR is slightly higher than the one of RR-ROIG but the results are comparable. This newly presented online RR-ROIG would thus be valuable when fewer dimensions are required to be fed to the learning algorithm. This would be the case, for instance, in the presence of a high number of sensors and when there would be a CPU limitation. This is clearly advantageous when the software will be transferred to a prosthesis-embedded system in the future.

Our experiment was also tested by one trans-radial amputee. His results in terms of success rate are relatively low compared to the ones of the intact subjects. However, in each subgroup of types of combined movement, half or more of the tasks were reachable (without achievement of the 1.5 s in task) at least once during the 3 repetitions. Moreover, the TIT for successful tasks was around 2 s, which is comparable to the one for intact subjects (cf. Figure 11). As for intact subjects, the time to complete the tasks seems to increase with the complexity of the task, from “single actions” through “combined actions without the power grasp” to “combined actions including the power grasp,” indicating that the tasks became harder to achieve for the amputated subject as well as for the intact subjects. The TCT are nonetheless around 1.5 s more for the amputee, the tasks seemingly being harder to complete for him. However, as it can be seen in the complementary video, the subject was able to control the 3D hand model relatively well. Several possibilities could explain his low success rates. The first one being that the foam softness might not have been adapted to the relatively weak muscles of an amputee's stump and the amputated bulge could not create enough depth print on the tactile bracelet. We also speculate that the highly precise tasks of the TAC test were difficult to achieve for the subject. Lastly, we wanted the experiment with the amputee to be as similar as possible as the one with intact subjects. Therefore, we had the same length of familiarization phase. However, considering the impairment of the subject as well as the fact that he was not versed into technology, a longer familiarization phase might have improved the results. We are aware of the limitations of this study considering that we only tested one amputated person and, in the future, we want to test the device on more amputees after improving this prototype version. This study is, however, a first step in showing that this technique of TMG could also be used for the amputees.

One further limitation that we would like to address is the drift of the tactile bracelet, especially considering that it affected the experiment design with the necessity of retraining in between the 3 repetitions of the task reaching phase. Though hypothetically partially based on the elasticity of the skin, we suppose that the main part was coming from the bracelet itself and more specifically the foam that we used over the taxels. We tested several foams to counter this issue, including some harder foams bringing less drift but impairing the detection of the slight changes in the muscle pressure signature. However, we did not yet come to a satisfying definitive solution and are therefore investigating new innovative conductive materials.

## 6. Conclusion

In this study on 12 intact subjects, we demonstrated the feasibility of using a new technology called tactile myography for combined control of 2 combined DoFs in highly complex online TAC-test with simultaneous control of 3 DoFs (6 actions trained including rest), instead of the configuration of 2 DoFs, typically used in the literature. This control was achieved using simple ridge regression and an intuitive mapping. Performance degradation was however observed when including the power grasp into the combined movements. This limitation might yet be due to the tactile bracelet and further work on it will help us clear this point. In this study, we show that TMG is a viable alternative to EMG as a sensing device for gesture recognition. As a first step toward prosthesis control, we tested it on one amputee but the bracelet still needs improvements before further tests on more amputees are reasonable. TMG requires less electronics for the same resolution and is easily wearable, in comparison to the bulky EMG sensors, which additionally are prone to be influenced by sweating and muscle fatigue. For these reasons, TMG is a desirable alternative to the standard sEMG. On another note, by this experiment we show that despite non-linear algorithms being a solution to combined control over multiple DoFs, they might not be the optimal solution and a higher dimensionality of eventually different sensors could be a different path to follow that would be less cumbersome for the machine learning algorithm. Additionally, we proposed a feature selection method, selecting a lower number of sensors, that yields similar results than with the full set of sensors. We can reasonably argue that it could thus be a possible solution for embedding into a prosthesis, where power computation comes into play. Finally, we propose a set of combined motion limitations that can be re-used in similar experiments.

### Future Work

In the future, further work has to be done to try to improve the tactile bracelet prototype by increasing its depth resolution and having it adapt to the conic shape of the stumps. Despite already several trials, the thickness and the rigidity of the foam are still not optimal and we are working on fixing these issues by replacing the foam with a different material. Further experiments could include a test on the number of sensors needed to achieve the control of untrained combined actions, as well as a TAC test with three combined actions — since the set of limits previously defined already includes 3-DOF combinations. Moreover, in the advent of deep learning and with the high density of the sensors that we here have, different algorithms could be tested on this bracelet (e.g., de Freitas et al., 2019). Finally, it would be interesting to compare TMG with HD-EMG in terms of single and combined actions.

One last remark about the limitations of the present study: The problem of upper-limb prosthetics is a paradigmatic multi-disciplinary issue and needs focus from such diverse fields as, e.g., material science for the socket, movement science for the ergonomy and physiology of the apparatus as a whole, mathematics and mechatronics for the device and the control systems, and statistics to measure the acceptance in the patient population. In this paper we have focused on a promising device and approach, which lets us foresee a more-than-sEMG myocontrol system embedded in a prosthetic socket. Of course, as already mentioned in, e.g., Cho et al. (2016), tactile myography is subject to different hurdles and problems as sEMG, and they need to be taken into account, too. Moreover, a careful study of further alternatives to tactile myography is required [a promising one being ultrasound A-mode scanning Yang et al. (2020)], as well as a study on how the limb-position effect Betthauser et al. (2018) affects it. In the end, extensive tests on the end-user population are necessary.

## Data Availability Statement

The datasets generated for this study are available on request to the corresponding author.

## Ethics Statement

The studies involving human participants were reviewed and approved by Work Ethical Committee of DLR. The patients/participants provided their written informed consent to participate in this study. Written informed consent was obtained from the individual(s) for the publication of any potentially identifiable images or data included in this article.

## Author Contributions

Conceptualization, methodology, validation, and interpretation of data: CC and MC. System design: RK. Software design, formal analysis, and investigation: MC. Resources, supervision, project administration, and funding acquisition: CC. Writing—original draft, writing—review and editing: MC, RK, and CC. Visualization: MC and RK.

### Conflict of Interest

The authors declare that the research was conducted in the absence of any commercial or financial relationships that could be construed as a potential conflict of interest.

## Footnotes

^1^http://www.coaptengineering.com/

^2^https://www.youtube.com/watch?v=B8Z_2tMUeiw

^3^With a slight abuse of language, we will consider a power grasp as one single DoF along this article.

^4^All muscle's abbreviations are the standard ones and are explained in the abbreviation section.

## Abbreviations

APL: Abductor Pollicis Longus
CPU: Central Processing Unit
DoF: Degree of Freedom
ECRB: Extensor Carpi Radialis Brevis
ECRL: Extensor Carpi Radialis Longus
ECU: Extensor Carpi Ulnaris
ED: Extensor Digitorum
EDM: Extensor Digiti Minimi
EPL: Extensor Pollicis Longus
FCU: Flexor Carpi Ulnaris
FCR: Flexor Carpi Radialis
FDP: Flexor Digitorum Profundus
FDS: Flexor Digitorum Superficialis
FMG: Force Myography
FPL: Flexor Pollicis Longus
HD-FMG: High-Density Force Myography
MVC: Maximum Voluntary Contraction
ROI: Region Of Interest
ROIG: Region Of Interest Gradient
ROM: Range of Motion
RR: Ridge Regression
RR-ROIG: Ridge Regression with Region Of Interest Gradient
sEMG: Surface Electromyography
SD: Standard Deviation
SR: Success Rate
TAC: Target Achievement Control
TCT: Time to Complete Task
TIT: Time in Task
TMG: Tactile Myography
